# The Anatomy of the Nasal Cavity and Its Accessory Sinuses

**Published:** 1895-09

**Authors:** 


### Reviews of Books.

*The Anatomy of the Nasal Cavity and its Accessory Sinuses.* By DR. A. ÓNODI. Translated from the Second Edition by ST. CLAIR THOMSON, M.D. London: H. K. LEWIS. 1895.

These plates have been prepared from a series of sections through the nasal cavities, made by Dr. A. Ónodi, of Buda Pesth. The dissections have been first photographed and then engraved, so that each figure is perfectly true to nature and is not produced by combining in one picture the results of several dissections. The result is that the topographical relations of the nasal fossæ are rendered easy to understand, and by comparing vertical, coronal, and horizontal sections we can obtain as clear an idea of the intricacies of these parts as is possible by means of illustrations. The work is worthy of the highest praise. Each plate is abundantly explained by references on the plate itself, while a fuller description is given in the introduction. We are rather surprised to notice that in Plate 3 the author describes the superior meatus as comprising not only the part between the superior and middle turbinated bones, but also the part above the former, between it and the roof of the nose. This is generally described in English text-books as the sphenoethmoidal recess, and the sphenoidal sinus is described as opening here instead of into the superior meatus as given by Dr. Onodi.

The plates will form a valuable means of instruction to anatomists and rhinologists.

